# The Effect of Oxygen Enrichment on Cardiorespiratory and Neuropsychological Responses in Workers With Chronic Intermittent Exposure to High Altitude (ALMA, 5,050 m)

**DOI:** 10.3389/fphys.2018.00187

**Published:** 2018-03-23

**Authors:** Fernando A. Moraga, Iván López, Alicia Morales, Daniel Soza, Jessica Noack

**Affiliations:** ^1^Laboratorio de Fisiología, Hipoxia y Función Vascular, Departamento de Ciencias Biomédicas, Facultad de Medicina, Universidad Católica del Norte, Coquimbo, Chile; ^2^Safety Group, Atacama Large Millimeter Submillimeter Array, Calama, Chile; ^3^Departamento de Ciencias de la Salud, Escuela de Enfermería, Universidad Santo Tomás, Santiago, Chile

**Keywords:** oxygen enrichment, neuropsychological impairment, oxygen saturation, heart rate, arterial pressure, chronic intermittent hypobaric hypoxia

## Abstract

It is estimated that labor activity at high altitudes in Chile will increase from 60,000 to 120,000 workers by the year 2020. Oxygenation of spaces improves the quality of life for workers at high geographic altitudes (<5,000 m). The aim of this study was to determine the effect of a mobile oxygen module system on cardiorespiratory and neuropsychological performance in a population of workers from Atacama Large Millimeter/submillimeter Array (ALMA, 5,050 m) radiotelescope in the Chajnantor Valley, Chile. We evaluated pulse oximetry, systolic and diastolic arterial pressure (SAP/DAP), and performed neuropsychological tests (Mini-Mental State examination, Rey-Osterrieth Complex Figure test) at environmental oxygen conditions (5,050 m), and subsequently in a mobile oxygenation module that increases the fraction of oxygen in order to mimic the higher oxygen partial pressure of lower altitudes (2,900 m). The use of module oxygenation at an altitude of 5,050 m, simulating an altitude of 2,900 m, increased oxygen saturation from 84 ± 0.8 to 91 ± 0.8% (*p* < 0.00001), decreased heart rate from 90 ± 8 to 77 ± 12 bpm (*p* < 0.01) and DAP from 96 ± 3 to 87 ± 5 mmHg (*p* < 0.01). In addition, mental cognitive state of workers (Mini-Mental State Examination) shown an increased from 19 to 31 points (*p* < 0.02). Furthermore, the Rey-Osterrieth Complex Figure test (memory) shown a significant increase from 35 to 70 (*p* < 0.0001). The results demonstrate that the use of an oxygen module system at 5,050 m, simulating an altitude equivalent to 2,900 m, by increasing FiO_2_ at 28%, significantly improves cardiorespiratory response and enhances neuropsychological performance in workers exposed to an altitude of 5,050 m.

## Introduction

Exposure to high altitude has become an increasingly common event. As many as 140 million people live at altitudes over 2,500 m (Moore, 2001). However, mining activities at altitudes over 3,500 m are common in in the north of Chile and Peru. In Chile up until 1995, there were approximately 20,000 workers intermittently exposed to high altitudes for a long period of time (Jiménez, 1995), and this number is expected to increase to over 120,000 by the year 2020 (http://www.ccm.cl/wp-content/uploads/2016/06/fuerza_laboral_de_la_gran_mineria_chilena_2012_2020.pdf). In these conditions, chronic intermittent hypobaric hypoxia (CIHH) constitutes a model of hypobaric hypoxic exposure previously described by several authors (Jiménez, 1995; Richalet et al., 2002; Moraga et al., 2014).

The physiological consequences of the initial exposure to high altitude are widely known. For example, the incidence of acute mountain sickness depends on altitude reached, previous experience, velocity of ascent and is self-limited by exposure time (Davis and Hackett, 2017). Early studies showed the effect of acute exposure on neuropsychological performance. For instance, the reduction in oxygen at high altitude impairs mental and physical performance, and general well-being (Barcroft et al., 1923). In addition, studies performed in a population of miners at high altitude showed deterioration in cognition and motor function (Mc Farland, 1937). Exposure to high altitude resulted in impaired sleep quality with increased periodic breathing, increased awakenings and shorter stage 3 and 4 sleep, causing low productivity and altered general well-being (Gerard et al., 2000). All these effects were dependent on the altitude attained.

One way to avoid the consequences of high altitude hypoxemia is to reduce the equivalent altitude (altitude which provides the same PO_2_ in moist inspired gas during ambient breathing). The first physiological response is given by an increase in ventilation and second by an artificial increase in oxygen supply. Therefore, we evaluated two different procedures that provide supplemental oxygen at high altitudes: an oxygen concentrator (West, 2016) and the use of liquid oxygen (Moraga et al., 2014). Both procedures mimic the higher oxygen partial pressure of a lower altitude by increasing in the oxygen fraction. The beneficial effects of this procedure enhancing sleep quality, neuropsychological function, and arterial oxygenation has been demonstrated in subjects exposed to conditions of simulated hypoxia at sea level, and subjects that have been acutely exposed to altitude hypoxia (West, 1995; Luks et al., 1998; Gerard et al., 2000; McElroy et al., 2000). Currently the only study performed during exposure to high altitude at 4,200 m, in miners acclimatized to CIHH, evaluated sleep quality and the nocturnal ventilation pattern, finding that they were significantly enhanced by oxygen administration (Moraga et al., 2014). However, no studies have evaluated a CIHH population at altitudes over 4,200 m. In addition, the ministry of health provided a technical guide for high altitude workers in 2014 that included a series of recommendations to reduce malaise during exposure to altitudes over 3,000 m. In regards to environmental oxygenation, the oxygen equivalent must be below 3,000 m with permanent control of temperature, relative humidity and room ventilation (web.minsal.cl/sites/default/files/guia_hipobaria_altitud.pdf). Therefore, our aim was probe a mobile module that reduced the equivalent altitude to values of 2,900 m, in an isolated sector at a very high altitude of 5,050 m in a population of workers from the Atacama Large Millimeter/ submillimeter Array (ALMA) radiotelescope in the Chajnantor valley, (Chile) and evaluate cardiorespiratory and neuropsychological on workers in both conditions.

## Subjects and methods

### Subjects

Thirteen voluntary subjects that lived at a low altitude (<1,000 m) and worked at ALMA (2,900 and 5,050 m) were studied. All subjects worked as antenna operators and had experience with CIHH for more than 4 years. Their shift pattern was 8 days of work at high altitude followed by 6 days rest at sea level. All subjects were free of cardiovascular, pulmonary, hematological, renal or hepatic diseases. All protocols and procedures performed in the present study were performed by following the Helsinki guidelines and have previously been approved by the Ethics Committee of the Facultad de Medicina of Universidad Católica del Norte and the ALMA Safety Department. A consent informed was obtained in written form each one of them previous being monitored.

### Facilities

ALMA is located 30 Km from the town of San Pedro de Atacama. ALMA has two sectors: the first is located 16 kilometers from the town of San Pedro de Atacama and represents the base camp or Operations Support Facility (OSF) at 2,900 m; and the second sector is the Array Operation Site (AOS) where the antennas are located in the Chajnantor Valley. This valley is located 40 km from OSF at an altitude of 5,050 m. The full personnel capacity of ALMA (OSF and AOS) is close to 400 people, including scientists, operation specialists, and services that operate in chronic intermittent hypobaric hypoxia exposure.

### Equipment

**Figure 1:**
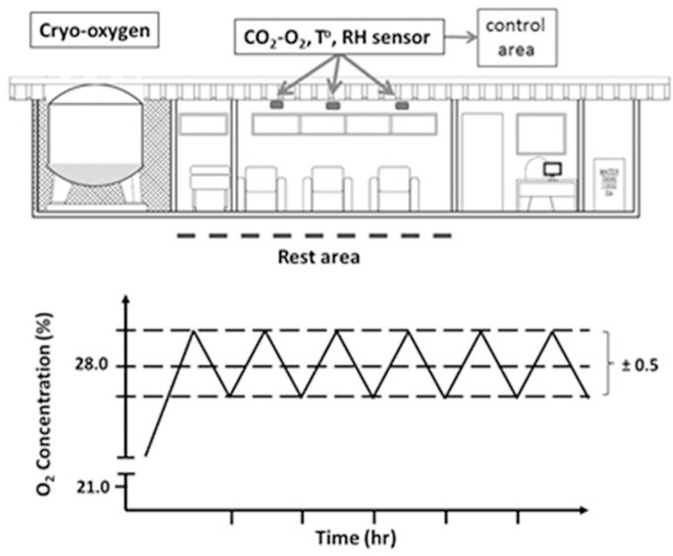
The scheme represents a diagram of the mobile unit composed of three areas: cryo-oxygen, rest and control area. Control area measured concentration of gases (O_2_ and CO_2_), relative humidity (HR), and temperature (°C) and maintained these values at the established requirements. The bottom graph represents the oxygen level control pattern at 5,050 m.

This study was performed at the AOS at 5,050 m where we designed a comfortable and mobile module (Oxymind™, INDURA, Chile) with facilities to increase the oxygen concentration (1 to 10%) and enhance the relative humidity and temperature (Figure 1). The oxygen concentration of the room air was controlled by a wide range oxygen sensor (0–50%, accuracy ± 2 ppm; Ultima XA, MSA, Pittsburg, USA) and maintained by a precise servo control system that allowed for an increase in oxygen concentration in 7% to reach 28 ± 0.5%, representing an equivalent altitude of 2,900 m with a lower low fire risk (West, 2001, 2003). In addition, precise control of CO_2_, by using an infrared CO_2_ sensor (ppm or % volume) (model Eagle 2, RKI Instruments Inc., Buffalo Grove, USA), was obtained by activating a fan (near 1400 m^3^/h) in order to reduce the accumulation of CO_2_ in the room (with a maintenance level of CO_2_ < 0.1%). Therefore, ventilation of the room was maintained by following the procedure described by West (1995) and Luks et al. (1998).

### Study protocol

Cardiorespiratory responses and neuropsychological evaluations were performed in acclimatized workers 48 h after arrival to high altitude. All parameters were measured in two conditions: base camp (OSF, 2,900 m) and AOF (5,050 m) where voluntaries were evaluated breathing ambient air and breathing room air enriched with oxygen (28 ± 0.5%) at 5,050 m in order to reduce equivalent altitude to 2,900 m. The total exposure time in each condition was 20 min (20 min for cardiovascular and neuropsychological evaluations).

### Cardiorespiratory evaluations

Heart rate and oxygen saturation were continuously recorded in the subjects during the procedures with a pulse oximeter (Wristox 3100, Nonin, Minnesota, USA), all recordings were analyzed by nVision software (Nonin, Minnesota, USA). SpO_2_ and heart rate values were extracted and analyzed min-min. Systolic arterial pressure (SAP), diastolic arterial pressure (DAP) and mean arterial pressure (MAP) were taken during the neuropsychological evaluation (model BM3, Bionet).

### Neuropsychological evaluation

In order to evaluate the effect of oxygen supply on cognitive function, a neuropsychological test was given to each volunteer. This test covered cognitive functions for memory and constructive praxis (Rey-Osterrieth Complex Figure test and Mini-Mental State Examination, MMSE).

### Rey-osterrieth complex figure test

In our study, we use one of the most widely used test in both clinical and experimental settings to evaluate visuoconstructional abilities and nonverbal memory (Frank and Landeira-Fernandez, 2008). Subjects were given a blank paper to draw the figure best. They don't have of time for the copy or process to memory recall the draw. Afterward, the subjects were evaluated cardiorespiratory variables and MMSE, the time frame for this evaluation was approximately 10 min, and without warning each subject was given another blank sheet to recall the figure. The total time of evaluation, is don't was major than 15–20 min, in order to reduce the effects of fatigue produced by prolonged neurocognitive tests. Furthermore, time over 30 min the performance decay of this time (Loring et al., 1990). Each drawing was evaluated using a traditional scoring unit for the complex figure of 18 particular items of the complex figure (Loring et al., 1988), where each particular item was evaluated in accord to a two-point, with a total score of 36 points (Table 1). Considering that in our country we don't have a cut off values. However, empirical experience is accepted to copy values <27 points and memory values <17 points to both condition was considered such as deficiency visuoconstructional abilities and no verbal memory, respectively.

**Table 1:** Traditional evaluation scoring for Rey-Osterrieth complex figure.

**Criterias by each item**	**Score (0–2)**
Placed and reproduced correctly	2
Reproduced incompletely placed incorrectly or presented some distortion	1
Placed or reproduced poorly	0.5
Absent or not recognized	0

*Frank and Landeira-Fernandez (2008)*.

### Mini-mental state examination (MMSE)

In our study we use the MMSE version of 35 points (Folstein et al., 1975). Each subject was examined in 5 mental state: Orientation (10 points), Registration (3 points), Attention and calculation (8 points), Recall (3 points) and language (12 points). The presence of subject with values <24 points were considered cognitive alteration.

All analysis of each drawing of Rey-Osterrith Complex Figure test and MMSE were performed by blind evaluator at sea level. The lower neuropsychological test selection for our study was given by empirical experiences; these tests were significant at high altitude in workers with CIHH at 3,500 and 4,600 m (unpublished data).

### Statistical analysis

All results were expressed as mean ± standard deviation. Cardiorespiratory variables (SpO_2_, HR, SAP, DAP) differences on means were analyzed using ANOVA followed by a Newman-Keuls test. Wilcoxon (Non-parametric test) to paired comparison was used to compare the difference of neuropsychological test. All differences were considered statistically significant when *p* < 0.05. Data analyses were performed using GraphPad Prism version 5.03 (GraphPad Software, Inc.).

## Results

### Cardiorespiratory variables at 2,900 and 5,050 m

**Figure 2:**
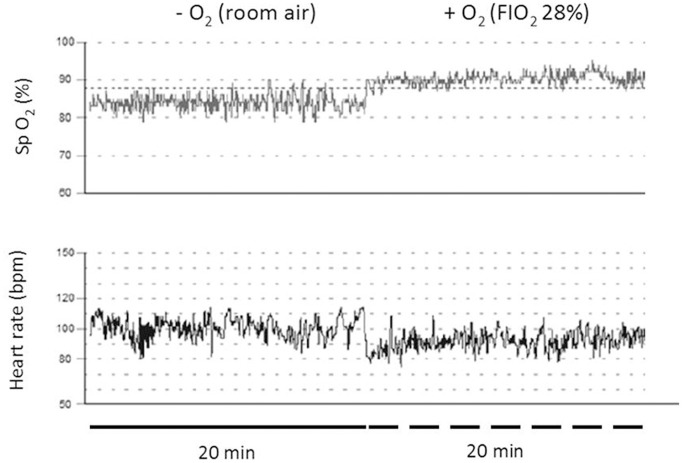
Representative records of subjects tested at Chajnantor valley's (5,050 m) when breathing air alone (thin line pulse oximetry and thick line heart rate). The thick and segmented lines represent recording of 20 min without and with oxygen, respectively.

Table 2 show a summary of the cardiorespiratory response obtained at camp base (OSF at 2,900 m) before ascending to the AOS at 5,050 m and cardiorespiratory values obtained in the AOS at 5,050 m. Pulse oximetry was decreased to values of 84 ± 0.8% (*p* < 0.05) that represent a fall approximately 9.7% in the arterial oxygenation and heart rate was increased to values of 90 ± 8 bpm (*p* < 0.05) that represented an increase of 16.8%, systolic arterial pressure was increased to values of 135 ± 18 mmHg that represented an increase of 11.6%, diastolic arterial pressure was increased to values of 96 ± 3 mmHg (*p* < 0.05) that represented an increase of 18.5% and mean arterial pressure was increased to values of 109 ± 8 mmHg (*p* < 0.05) that represented an increase of 15.9% was observed in all subjects when they arrived at 5,050 m, respect of values obtained in camp base (OSF, 2,900 m). During the protocol at 5,050 m when the subjects breathed room air, no modifications to the cardiorespiratory variables were observed. However, when subjects were placed in the module with an increase in FiO_2_ to 28%, we observed a fast increase in the pulse oximetry to values of 91 ± 0.8 % (*p* < 0.05) that represented an increase of 8% arterial oxygenation and a fall in the cardiovascular response such as heart rate to values of 77 ± 12 bpm (*p* < 0.05) that represented a decreases by 14.4%, systolic arterial pressure to values of 128 ± 10 that represented a decreases 5.2%, diastolic arterial pressure to values of 87 ± 5 mmHg (*p* < 0.05) that represented a significant fall of 9.4% and mean arterial pressure to values of 101 ± 7 mmHg (*p* < 0.05) that represented a significant fall of 7.3%. No differences were observed in cardiorespiratory variables between OSF and AOS + O_2_, reaching values similar to those observed at 2,900 m (OSF). Figure 2 shows a typical pattern of oxygen saturation and heart rate recordings in a worker during a period of 40 min.

**Table 2:** Cardiorespiratory response of workers exposed at 2,900 and 5,050 m.

	**Altitude**		
	**2,900 m**	**5,050 m**	
	**Room air**	**Room air**	**Room air + 8 %O_2_**
SpO_2_ (%)	93 ± 1	84 ± 0.8^*^	91 ± 0.8^*^
Heart rate (bpm)	77 ± 8	90 ± 8^*^	77 ± 12^*^
SAP (mmHg)	121 ± 6	135 ± 18	128 ± 10
DAP (mmHg)	81 ± 6	96 ± 3^*^	87 ± 5^†^
MAP (mmHg)	94 ± 8	109 ± 8^*^	101 ± 7^†^

Values expressed as Mean ± SD. SpO_2_, pulse oximetry; SAP, systolic arterial pressures; DAP, diastolic arterial pressure; MAP, mean arterial pressure.

* p < 0.001 Room Air 2,900 vs. Room Air 5,050 m and 5,050 m + 8% O_2_,

† *p < 0.01 Room air 5,050 m vs. Room Air 5,050 m + 8% O_2_*.

### Neuropsychological assessment

**Figure 3:**
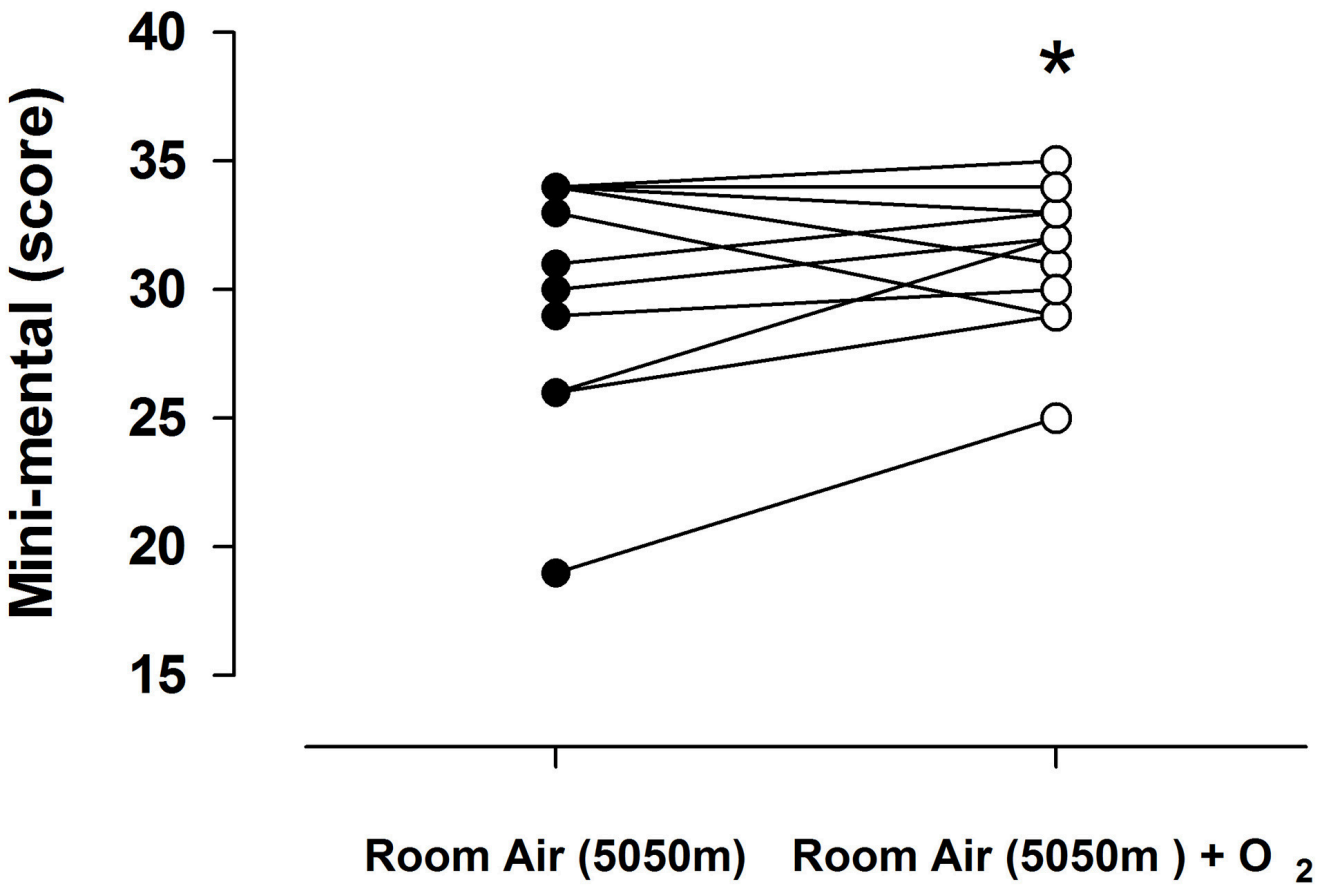
Individual response on minimental score (MMscore) in 13 subjects breathing room air of 5,050 m (open circles) and room air of 5,050 m enriched with oxygen (closed circles). Values represent the score obtained of each subject in both condition. Lines represent the mean ± SD, asterisk *p* < 0.001.

Neuropsychological evaluations were performed with MMSE and revealed that when the subjects were tested in environmental oxygen conditions, 30% of the population presented disorders. However, upon entering the module with oxygen enrichment, this was reduced to 10% (*p* < 0.05). Figure 3 represents the individual score obtained by MMSE.

**Figure 4:**
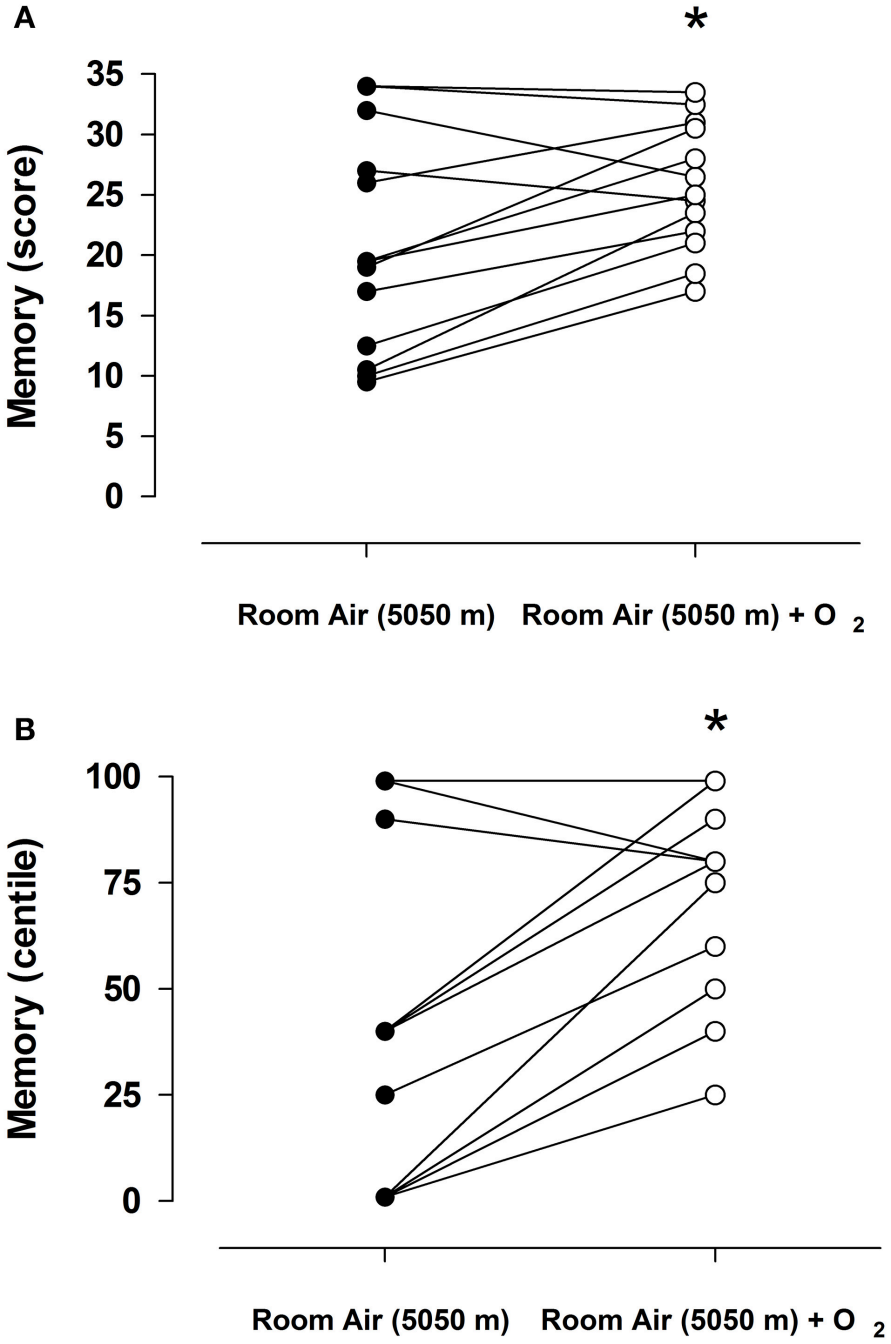
Individual response to the Rey-Osterrieth Complex Figure (memory) in 13 subjects breathing room air of 5,050 m (open circles) and room air of 5,050 m enriched with oxygen (closed circles). **(A)** Represent absolute score obtained of each subject in both condition, and **(B)** represent values expressed in centile. The lines represent the mean ± SD, asterisk *p* < 0.001.

When asked to evoke the Rey-Osterrieth Complex Figure test (memory), this capacity was significantly reduced in workers exposed to room air, with 50% of the patients meeting the criteria of a memory disorder (Figures 4A,B). However, when subjects were tested in the oxygen enriched module, the percentage of subjects below the cut off criteria was 10%, indicating a significant difference compared to subjects tested in environmental oxygen conditions.

## Discussion

This study is the first to assess the effect of environmental oxygen enrichment at 28% in very high altitude conditions (5,050 m) in a population of volunteers acclimatized to chronic and intermittent hypobaric hypoxia exposure for at least 4 years. Our results demonstrate that the mobile module that permits increased oxygen concentration, used to simulate an altitude equivalent to 2,900 m, significantly improved oxygen saturation and reduced heart rate and diastolic blood pressure, and enhanced neuropsychological performance in workers exposed to an altitude of 5,050 m.

Studies performed in CIHH models are scarce. Richalet et al. (2002) published results of a prospective study of workers exposed to CIHH for 2.5 years at an altitude of 4,500 m and described a developing acclimatization pattern along with emphasized health risks; right ventricular dilation and increased blood pressure. In addition, the incidence of acute mountain sickness is elevated on the first day, and is reduced, but does not disappear, with exposure time (Richalet et al., 2002). In addition, Brito et al. (2007) reported tachycardia, high blood pressure and low oxygen saturation after 12 years of CIHH exposure at 3,550 m. In addition, Siqués et al. (2009) found a significant increase in the diastolic pressure associated to lower oxygen saturation in young adults after 12 months of exposure at the same altitude. A study in acclimatized miners (1–14 years of CIHH exposure) showed the existence of periodic apnea breathing with episodes of arterial oxygen saturation reaching 67% (Moraga et al., 2014) at an altitude of 4,200 m. This severe level of hypoxemia triggers an increase in sympathetic tone. Previous studies support the observation that high altitude exposure promotes sympathetic activation and increases arterial pressure (Wolfel et al., 1994; Hansen and Sander, 2003). A preliminary study showed that 63% of people working at an altitude of 4,600 m had increased arterial pressure (>140/>90 mmHg) and oxygen saturation (<80%) (Moraga, unpublished results). In previous studies performed in our laboratory demonstrated that oxygen enrichment by 3–4% in a room at 4,200 m simulates an oxygen equivalent of 3,200 m, enhancing oxygen saturation, reducing heart rate, and inducing a general feeling of well-being in the morning. A better explanation of the effect of a decreased equivalent altitude with increased oxygen room is a decrease in the activation of the peripheral chemoreceptor as a consequence of reduced central neurosystem activation (see Dempsey et al., 2014). Previous studies support the role of carotid bodies in animal models where the carotid afferent was cut off, resulting in the abolishment of the sympathetic and hypertensive responses (see Prabhakar et al., 2012; Del Rio et al., 2016; Iturriaga et al., 2017).

In regard to the neuropsychological performance evaluated in our study, acute decreased oxygen availability at high altitudes is known to produce impaired mental and physical performance, increasing as the altitude also increases. The first studies performed in miners at 3,800 m, showed deterioration in cognition and motor function (Mc Farland, 1937). The first study that reported modifications in the neuropsychological response in miners exposed to CIHH, showed a decrease by 5% in the general cognitive aptitude on the 5th shift day at 4,500 m. The most affected elements were spatial aptitude (13% decrease) and mathematical reasoning (28% decrease) and neuropsychological attention (11% decrease) compared to evaluations performed at sea level (Jiménez, 1995). Additionally, in controlled hypobaric hypoxia conditions with an over imposed hypoxia of 5,000 m, neuropsychological function after increase of 6% percentage of oxygen on room, in no acclimatized voluntaries, reported an increase in oxygen saturation, quicker reaction times, improved hand-eye coordination, and more positive sense of well-being (Gerard et al., 2000). However, the study showed no significant improvement on neurophysiological function (Gerard et al., 2000). Therefore, our study is the first to assess neuropsychological performance in a population of acclimatized workers exposed to CIHH at a very high altitude (5,050 m) where room air levels were supplemented with oxygen at FiO_2_ 28% to obtain an equivalent altitude of 2,900 m. Exposure to high altitude decreased neuropsychological and cognitive functions for memory and constructive praxis (Rey-Osterrieth Complex Figure test, MMSE), however, this decreased response was reverted by increasing environmental oxygen at 28% in the room, supporting the notion that this response is due to low oxygen availability at very high altitudes, and our results suggest that this is a plastic response.

By other way, since the early work of Paul Bert in 1878, it has been assumed that the lower inspiratory pressure of oxygen is the primary stimulus for adaptation to hypoxia (See Conkin and Wessel, 2008). Later, Barcroft in 1925 showed that decreasing FIO_2_ could produce a hypoxic condition (See Richard and Koehle, 2012). These studies were the starting point for the discussion about whether the physiological responses are equivalent in normobaric hypoxia and hypobaric hypoxia models (Savourey et al., 2003; Richard and Koehle, 2012) in order to predict susceptibility to suffer from acute mountain sickness (Savourey et al., 2003; Burtscher et al., 2008), optimal training for athletes (Levine and Stray-Gundersen, 2006) and others. In a recent review, evidence supported a difference in ventilation mechanisms between both hypoxia models (Coppel et al., 2015). In addition, the same study showed that the confounding factors such as time spent, temperature, humidity and statistical power, limit the conclusion of these finding (Coppel et al., 2015). However, a series of studies have shown that the hypobaric conditions of high altitude could have physiological effects that are independent of the fall in oxygen pressure (i.e., temperature, air density, fluid balance, the presence of a microbubble in pulmonary circulation, increased dead space, etc.) (Loeppky et al., 2005; Conkin and Wessel, 2008; Richard and Koehle, 2012; Coppel et al., 2015); but it is necessary to indicate that many of these studies were performed in simulated conditions (hypobaric or normobaric chambers). These conditions are very different to field studies, like that performed in our study since this evaluates workers acclimatized to CIHH at very high altitude (5,050 m), where increasing FiO_2_ enhances well-being during their stay at high altitude. In a previous study, miners acclimatized to CIHH showed a different response to hemoconcentration during maximum exercise performed at sea level and high altitude (3,800 m), given by a mechanism mediated by a negative water balance (low intake, high loss) (Moraga et al., 2017), supporting the idea that the hypobaric condition of high altitude promotes a different response. Another, study evaluated heart rate and oxygen saturation during a hike at high altitude (Manua Kea, 4,205 m) and compared this with a simulate condition (normobaric hypoxia). The results showed that oxygen saturation was lower and heart rate was higher at high altitude compared with normobaric hypoxia (Netzer et al., 2017). More studies are necessary to improve our understanding of the mechanisms involved in tolerance and acclimatization to hypoxia, in all hypoxia exposure models (in our case, in miners exposed to CIHH).

## Limitations

In our study it was impossible to perform a crossover long term study in the workers as they were only authorized by their supervisors to participate in the first evaluation, due to the high labor demand during the study period.

In conclusion, our study showed that the use of a mobile module oxygen system at 5,050 m, simulating an altitude equivalent to 2,900 m, significantly improves arterial oxygenation and heart rate, reduces diastolic blood pressure, and enhances neuropsychological performance. Our data support the requirements that the health ministry should implement to enhance the environmental work place; preventing and reducing the risk of labor at altitudes over 3,000 m.

## Author contributions

FM conceived and designed the study. IL and AM supervises the overall the study. AM performed the statistical analysis. DS, AM, and JN contributed to sample and data collections. All authors drafted the report. All authors contributed to the interpretation of the results, critical revision of the manuscript and approved the final manuscript. FM is the guarantor.

### Conflict of interest statement

The authors declare that the research was conducted in the absence of any commercial or financial relationships that could be construed as a potential conflict of interest.
